# Draft Genome Sequences of Anaplasma marginale Strains MEX-15-099-01 and MEX-31-096-01, Two Mexican Isolates with Different Degrees of Virulence

**DOI:** 10.1128/MRA.01184-19

**Published:** 2019-11-07

**Authors:** Fernando Martínez-Ocampo, Rosa Estela Quiroz-Castañeda, Itzel Amaro-Estrada, Mayra Cobaxin Cárdenas, Edgar Dantán-González, Sergio Rodríguez-Camarillo

**Affiliations:** a Laboratorio de Estudios Ecogenómicos, Centro de Investigación en Biotecnología, Universidad Autónoma del Estado de Morelos, Cuernavaca, Morelos, Mexico; b Unidad de Anaplasmosis, Centro Nacional de Investigación Disciplinaria en Salud Animal e Inocuidad, INIFAP, Jiutepec, Morelos, Mexico; Broad Institute

## Abstract

Anaplasma marginale is an intraerythrocytic bacterium that causes bovine anaplasmosis and is endemic in Mexico. In this work, we report two draft genome sequences of Mexican isolates from different geographical regions and with different degrees of virulence.

## ANNOUNCEMENT

Anaplasma marginale is an intraerythrocytic rickettsial Gram-negative bacterium that causes clinical signs of bovine anaplasmosis, namely, fever, anemia, jaundice, weakness, and even respiratory distress. A. marginale is endemic in tropical and subtropical areas of the world, including Mexico (1). Only one genome sequence, that of the Mexican strain MEX-01-001-01 from Aguascalientes, has actually been reported (2). In 2000, García et al. evaluated the level of virulence of two Mexican strains, the high-virulence strain MEX-15-099-01 isolated from Texcoco, State of Mexico, and the low-virulence strain MEX-31-096-01 isolated from Tizimin, Yucatan (3). Also, in 2008, Rodríguez-Camarillo et al. reported that strain MEX-31-096-01 is used as a live vaccine (4). Here, we report the draft genome sequences of A. marginale strains MEX-15-099-01 and MEX-31-096-01, which were isolated from the blood of sick cattle and blood of asymptomatic cattle, respectively.

We used 200 μl of bovine blood for each isolate to extract genomic DNA using the UltraClean DNA BloodSpin kit (Mo Bio Laboratories). Genomic DNA (2 μg) for each isolate was sequenced using the MiSeq platform (Illumina), and the libraries were prepared by fragmenting the genomic DNA and ligating specialized adapters to both fragment ends (Arizona State University DNA Sequencing Core). We obtained two data sets of 605,370 (MEX-15-099-01) and 1,671,658 (MEX-31-096-01) 300-bp paired-end reads, which were reported to the SRA database.

The Illumina adapter sequences were removed from paired-end reads using the ILLUMINACLIP trimming step of Trimmomatic (version 0.36), with default settings (5). Low-quality bases were removed using the dynamictrim algorithm of the SolexaQA++ suite (version 3.1.7.1) (6) with a Phred quality score (Q) of <13. The resulting paired-end reads were *de novo* assembled using SPAdes (version 3.11.1) (7) with the following options: (i) only run the assembly module (-only-assembler), (ii) reduce the number of mismatches (-careful), and (iii) k-mer lengths between 21 and 127. Contigs of two Mexican strains were differentiated from contigs that belong to other organisms (i.e., bovine genomes) based on G+C content of each contig assessed using a Python script (https://github.com/FernandoMtzMx/GC_content_MultiFasta) (the A. marginale genomes reported in databases have a G+C content between 46 and 52%). Also, we aligned the sequences of each contig assembled with the NCBI Nucleotide (nr/nt) database using the BLASTN suite (8) and “Anaplasma marginale” as the organism name. Contigs with an alignment coverage of greater than 50% and an identity of greater than 70% belong to A. marginale genomes. The features of the two draft genomes were evaluated using QUAST (version 4.6.2) (9). The assembly statistics are shown in Table 1.

**TABLE 1:** Statistics of the two assembled genomes

Statistic	Value for strain:	
	MEX-15-099-01	MEX-31-096-01
Genome size (bp)	1,169,440	1,176,579
No. of contigs	32	43
*N*_50_ length (bp)	85,955	62,699
Coverage (×)	∼30	∼19
G+C content (%)	49.79	49.79
GenBank accession no.	VTWW00000000	VTWV00000000
No. of genes	1,185	1,204
No. of CDS^*a*^	1,145	1,164

a CDS, coding sequences.

The draft genomes of strains MEX-15-099-01 and MEX-31-096-01 were annotated automatically using the Rapid Annotations using Subsystems Technology (RAST; version 2.0) server (10). The annotation statistics are shown in Table 1. The 16S rRNA gene sequences of two Mexican strains were obtained using the RNAmmer server (version 1.2) (11), and each has a length of 1,491 bp, as well as 100% alignment coverage and 100% identity with the 16S rRNA gene sequence of A. marginale strain St. Maries (GenBank accession number CP000030).

### Data availability.

This whole-genome shotgun project has been deposited at DDBJ/ENA/GenBank under the accession numbers VTWW00000000 and VTWV00000000 for MEX-15-099-01 and MEX-31-096-01, respectively. The versions described in this paper are versions VTWW01000000 and VTWV01000000 for MEX-15-099-01 and MEX-31-096-01, respectively. The publicly available raw data numbers (SRA) are SRR10197851 and SRR10198112 for MEX-15-099-01 and MEX-31-096-01, respectively.
